# Predictive Role of Interleukin-18 in Liver Steatosis in Obese Children

**DOI:** 10.1155/2018/3870454

**Published:** 2018-04-26

**Authors:** Marta Flisiak-Jackiewicz, Anna Bobrus-Chociej, Eugeniusz Tarasów, Małgorzata Wojtkowska, Irena Białokoz-Kalinowska, Dariusz Marek Lebensztejn

**Affiliations:** ^1^Department of Pediatrics, Gastroenterology and Allergology, Medical University of Białystok, Białystok, Poland; ^2^Department of Radiology, Medical University of Białystok, Białystok, Poland; ^3^Department of Radiology, University Children Hospital, Białystok, Poland; ^4^Łomża State University of Applied Sciences, Łomża, Poland

## Abstract

**Introduction:**

Interleukin-18 (IL-18) is a proinflammatory cytokine associated with metabolic syndrome (MS). Nonalcoholic fatty liver disease (NAFLD) can be recognized as a feature of MS.

**Material and Methods:**

Serum IL-18 concentration was evaluated in serum of 108 obese children, determined with ELISA, and referred to degree of liver steatosis in USG or total intrahepatic lipid content assessed by magnetic resonance proton spectroscopy (^1^HMRS).

**Results:**

Fatty liver was confirmed in 89 children with USG and in 72 with ^1^HMRS. IL-18 concentration demonstrated significantly higher values in patients than in controls. Significant correlations between IL-18 and ALT, GGT, triglycerides, hsCRP, and the degree of liver steatosis were demonstrated. NAFLD children had significantly higher level of IL-18, ALT, GGT, HOMA-IR, waist circumference, and total lipids content in ^1^HMRS than other obese children. IL-18 level was also significantly higher in obese children with advanced liver steatosis. Measurement of serum IL-18 showed ability to differentiate children with fatty liver from those without steatosis.

**Conclusion:**

Elevated serum IL-18 concentration and its correlation with hepatocyte injury, systemic inflammation, and degree of liver steatosis support role in NAFLD pathomechanism. IL-18 can be considered to play a role in predicting advanced liver steatosis and fatty liver in obese children.

## 1. Introduction

Nonalcoholic fatty liver disease (NAFLD) nowadays becomes a leading cause of liver pathology and may be present in 20–30% of general population. Rising prevalence of NAFLD is also observed among children and adolescents, which is closely related to obesity epidemic worldwide [1, 2]. The histological spectrum of NAFLD ranges from simple steatosis to nonalcoholic steatohepatitis (NASH), where steatosis coexists with hepatic inflammation that can be accompanied by fibrosis. Long lasting NASH may progress to severe liver damage and can result in cirrhosis or even hepatocellular carcinoma [3, 4]. Development of NAFLD is strongly associated with visceral obesity and other components of the metabolic syndrome (MS) including dyslipidemia and insulin resistance (IR); that is why NAFLD is regarded as a hepatic manifestation of MS [5, 6]. The pathogenesis of this clinical condition has not been fully understood but usually is explained by a “two-hit” hypothesis [7, 8]. In this mechanism the first “hit” is associated with excess accumulation of lipids in the liver. The second “hit” can lead to progression to NASH through increase of oxidative stress, lipid peroxidation, imbalance of adipokines level, or release of toxic products. Recent studies have revealed more possible factors involved in the development and progression of NAFLD, such as dysregulated intestinal microbiota, mitochondrial dysfunction, abnormalities of iron metabolism, or increased fructose consumption [9–15]. Moreover there are growing evidences showing that NAFLD, as well as IR, is strongly related to low-grade systemic inflammation and probably therefore some mediators are released from obese fat tissue and additionally promoting hepatic and systemic inflammation [16–21]. For this reason we have focused on investigation of proinflammatory cytokine interleukin-18 (IL-18) and its possible association with the development and the potential progression of NAFLD through stimulation of hepatic inflammation and fibrosis. However the most recent studies demonstrate differences between pediatric and adult NAFLD in terms of prevalence, histology, diagnosis, and management [22].

IL-18 plays a crucial role in the inflammatory cascade. This molecule is a member of the IL-1 family of cytokines and it was initially described as an interferon-*γ*- (IFN-gamma-) inducing factor. IL-18 is secreted in many different cell types, including macrophages, endothelial cells, vascular smooth muscle cells, dendritic cells, Kupffer cells, and adipocytes [23, 24]. It is primarily synthesized as a precursor protein, pro-IL-18, which requires activation by caspase-1 cleavage into bioactive mature form. IL-18 is inactivated by specific binding protein, which results from a negative feedback mechanism in response to increased IL-18 production, protecting cells from accelerated proinflammatory activity [25, 26]. Previously published studies demonstrated elevated circulating levels of IL-18 in adult patients with metabolic syndrome and its components. There are several reports suggesting its usefulness as a marker of insulin resistance in type 2 diabetes and predictor of cardiovascular disease development [27–30]. Furthermore there is some evidence that plasma levels of IL-18 are elevated in adults with chronic liver disease and correlate with severity of the disease [31–33].

These findings were demonstrated in adults and led us to investigate whether there is an association between serum IL-18 concentration and NAFLD in obese children. Moreover we analyzed effect of steatosis degree on serum IL-18 levels to establish its possible predictive role for the disease progression in comparison to several other markers of hepatic injury, metabolic dysfunction, and systemic inflammation.

## 2. Materials and Methods

### 2.1. Patients

This study comprised a group of 108 consecutive obese children (84 boys and 24 girls) aged 7–17 years (mean age 12,8 years) admitted to our department because of suspected liver disease based on hepatomegaly and/or elevated alanine aminotransferase (ALT) activity and/or fatty liver features on ultrasound (USG) examination. Informed consent was obtained from all patients' parents. The protocol was approved by the local bioethics committee. Viral infection due to hepatitis C virus (negative testing for anti-HCV) and hepatitis B virus (negative testing for HBsAg), autoimmune hepatitis, selected metabolic liver diseases (Wilson disease, alpha-1-antitrypsin deficiency, and cystic fibrosis), and drug-induced liver injury (DILI) were excluded in all children. Moreover, children with diabetes (elevated fasting plasma glucose) and those having infectious diseases on admission (white blood cell (WBC) count less than 3500 cells/*μ*l or more than 10 500 cells/*μ*l; C-reactive protein (CRP) more than 5 mg/L; body temperature more than 38°C) were excluded from this study.

All participants underwent a detailed medical history and physical examination with anthropometric measurements, including weight, height, body mass index (BMI), and waist circumference. BMI was calculated by dividing weight in kilograms by the square of height in meters (kg/m2). Obesity was defined as a BMI over the 95th percentile based on age and gender [34]. The diagnosis of NAFLD was established in children with both elevated serum ALT activity and liver steatosis on ultrasound examination. Liver steatosis was diagnosed with positive imaging results. Patients were divided into two groups: with NAFLD (*n* = 39) and obese without NAFLD (*n* = 69) that did not fulfil criteria of NAFLD diagnosis. All patients with elevated ALT had steatosis, but there were 50 patients with steatosis and normal ALT.

Age matched control group comprised of 15 nonobese children without any somatic organ pathology was recruited at the time of the study and blood samples were collected as a part of their routine checkup to obtain normal values of IL-18.

### 2.2. Laboratory Measurements

In all patients routine biochemical measurements were performed, including alanine aminotransferase (ALT), aspartate aminotransferase (AST), gamma glutamyltransferase (GGT), bilirubin, total cholesterol, high-density lipoprotein (HDL-C) and low-density lipoprotein (LDL-C) cholesterol, triglycerides (TG), uric acid, ferritin, glucose, and insulin, by using the enzymatic colorimetric method at laboratory department of our hospital. In all patients alpha-1-antitrypsin (A1AT) level and in 45 patients high-sensitive C-reactive protein (hsCRP) level were determined by a nephelometric method with a DADE Behring Nephelometer Analyzer (Germany).

Insulin resistance (IR) was calculated by homeostasis model assessment of insulin resistance (HOMA-IR) using the following formula: HOMA-IR = fasting insulin (*μ*U/ml) × fasting plasma glucose (mmol/L)/22.5 [35].

Serum IL-18 levels were determined by an enzyme-linked immunosorbent assay (Human IL-18 ELISA Kit, Medical & Biological Laboratories Co., Ltd., Nagoya, Japan). Samples were centrifuged and frozen in −80°C until further analysis. The minimum detection limit for IL-18 was 12.5 pg/ml. The intra- and interassay CVs of IL-18 ranged from 4.9 to 10.8% and 5.2 to 10.1%, respectively. The assays were conducted according to the manufacturer's instructions.

### 2.3. Liver Examination

Liver ultrasound examination was performed with Voluson E8 instrument (General Electric, USA), equipped with convex sonde 3–5 MHz. The degree of liver steatosis was graded using a four-grade scale (0–3) according to Saverymuttu et al. [36]. Advanced liver steatosis was defined as a score > 1. Steatosis grade was assessed by the same radiologist without knowledge of the patients' data. Proton magnetic resonance spectroscopy (^1^HMRS) was performed with a 1.5 T scanner (Picker Eclipse) and with PRESS sequencing. Total intrahepatic lipid content was assessed in relative units (r.u.) in comparison to unsuppressed water signal. Voxel's size 3 × 3 × 3 cm (27 cm3) was localized in the right liver lobe to avoid vessels and bile ducts [37].

### 2.4. Statistical Analysis

Results are reported as median, 25–75 quartiles. The statistical analysis was evaluated using the Mann–Whitney two-sample test for nonparametric data. The relationship between serum IL-18 concentration and variables was analyzed by the Spearman rank-correlation test for nonparametric data. Statistical significance level was set at *p* < 0.05. Logistic regression analysis was performed using IBM SPSS Statistics 20.0. The receiver operating characteristics (ROC) analysis was used to calculate the power of IL-18 to detect liver steatosis on ^1^HMRS. The comparison of the area under curve (AUC) was performed using a two-tailed *p*-test, which compares the AUC to the diagonal line of no information (AUC 0.5).

To perform combined ROC analysis of multiple parameters, linear combination of logistic regressions was carried out to detect children with liver steatosis in ^1^HMRS.

## 3. Results

Eighty-nine children (82,5%) had liver steatosis in ultrasound examination and 72 (78,2%) in ^1^HMRS with concordance rate of 100%. Moreover thirty-nine had also an elevated serum ALT activity (NAFLD patients).

Median IL-18 concentration measured in all obese patients (309; 237–410 pg/mL) was significantly (*p* = 0.038) higher than in controls (242; 197–318 pg/mL). As demonstrated in Table 1 median IL-18 level was significantly higher in NAFLD patients than in other obese children (*p* = 0.034). Moreover, patients with NAFLD showed significantly higher values of ALT, AST, and GGT activities, insulin, HOMA-IR, uric acid, ferritin, BMI, waist circumferences, degree of liver steatosis (USG), and total amount of lipids in ^1^HMRS compared to children without NAFLD (Table 1).

Significant positive correlations of IL-18 with ALT (*r* = 0.2, *p* = 0.036), AST (*r* = 0.21, *p* = 0.032), GGT (*r* = 0.23, *p* = 0.016), TG (*r* = 0.21, *p* = 0.027), hsCRP (*r* = 0.36, *p* = 0.014), and the degree of liver steatosis (USG) (*r* = 0.2, *p* = 0.044) were found (Table 2). Patients with steatosis in USG and elevated ALT demonstrated significantly higher IL-18 activity than those with steatosis but normal ALT (310 versus 339 pg/mL; *p* = 0.044). However it was not a case in patients with steatosis diagnosed using ^1^HMRS (341 versus 349 pg/mL;* p*-0.064).

**Figure 1:**
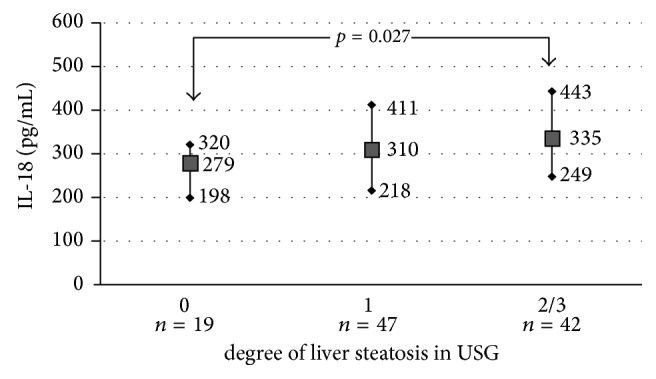
Median (min., max.) of IL-18 serum concentration depending on degree of liver steatosis in USG.

Among 108 obese children included in the study, 19 had no steatosis in USG (grade 0), 47 (44%) demonstrated moderate steatosis (grade 1), and 42 (39%) had advanced liver steatosis defined as grade 2 or 3 (Table 3). The concentration of IL-18 was significantly higher in obese children with advanced liver steatosis compared to children without steatosis (*p* = 0.027) (Figure 1).

**Figure 2:**
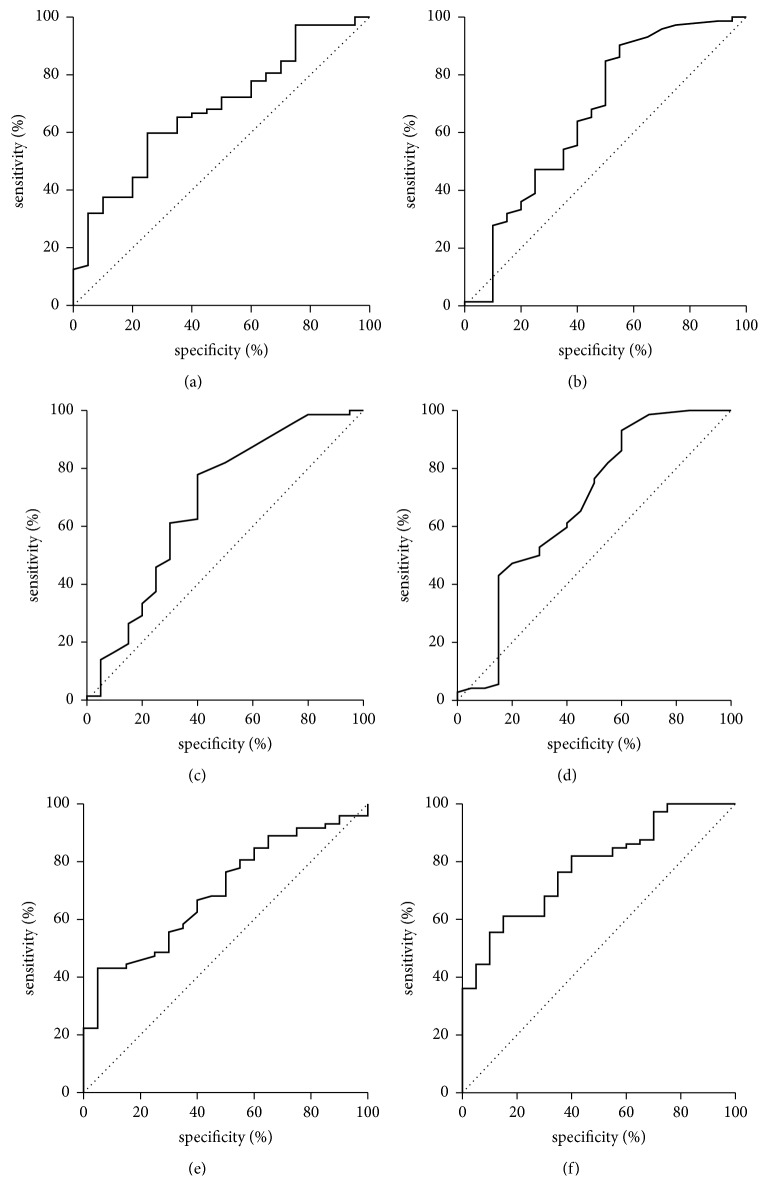
ROC curve for ability of IL-18 (a), ALT (b), AST (c), GGT (d), TG (e), and their combination (f) to detect children with liver steatosis in ^1^HMRS.

As shown in Table 4 children with liver steatosis measured with ^1^HMRS had significantly higher levels of IL-18, ALT, AST, GGT, TG, and intensity of liver steatosis in USG examination than those without liver steatosis in ^1^HMRS. In the logistic regression analysis IL-18 (OR-1.006, 95% CI for OR—1.001–1.010, and *p* = 0.018) and triglycerides (OR-1.015, 95% CI for OR—1.003–1.028, and *p* = 0.012) were the only measures useful in the differentiation of obese patients with liver steatosis confirmed using ^1^HMRS. This finding was confirmed in ROC analysis, which demonstrated a cut-off of IL-18 concentration on the level of 326.8 pg/ml as effective (AUC = 0.68; *p* = 0.006) for differentiation between children with or without fatty liver in ^1^HMRS (Table 5). Sensitivity was 60% and specificity 75%, whereas negative and positive predictive values were 34% and 90%, respectively (Figure 2(a)). ROC curves for ALT, AST, GGT, and TG demonstrate similar pattern to IL-18 (Figures 2(b), 2(c), 2(d), and 2(e)) as well as AUC values (Table 5). Combined ROC analysis of all five parameters was carried out to find out possible superior measure for steatosis corresponding to intrahepatic lipid content evaluated with ^1^HMRS. ROC analysis of combined parameters demonstrated superior AUC of 0.7826 (*p* < 0.0001), sensitivity 61%, specificity 85%, and negative or positive predictive value of 38% and 94%, respectively. However the ROC of the combined logistic regression model did not demonstrate statistical significance compared to individual AUC (Figure 2(f), Table 5).

## 4. Discussion

Early diagnosis of NAFLD in obese children is important for possible prediction of further metabolic disorders, which was supported in our study through significantly higher insulin concentration and HOMA-IR values related to insulin resistance. In this group of obese children with diagnosed NAFLD, we demonstrated significantly higher IL-18 serum concentration compared to both healthy controls and non-NAFLD obese children. This is in line with results from the study of Vecchiet at al. [33] who demonstrated higher IL-18 in NAFLD adult patients compared to controls but lower IL-18 than in patients with chronic hepatitis C, indicating possible role of hepatic inflammation in addition to steatosis regarding IL-18 production. The significant positive correlations between serum IL-18 and ALT and other indices of hepatocytes injury as well as hsCRP shown in our study support this hypothesis. However, we were not able to demonstrate significant elevation of hsCRP in any group, because its measurement was carried out in limited number of patients.

Contrary results with no effect on IL-18 in the absence of metabolic risk factors were demonstrated by Tapan et al. [38], but the study was conducted in male adults with NASH. IL-18 is a proinflammatory cytokine that can be produced by adipocytes, but also macrophages and Kupffer and endothelial cells [23, 24]. Therefore serum IL-18 production can be released as a result of inflammatory activity related to numerous concomitant health conditions, which obviously are much more frequent in adults than in children. Since children are less likely to have concomitant proinflammatory conditions than adults, measurement of IL-18 concentration can be useful for NAFLD diagnosis in children. In this study we were able to demonstrate significant increase of IL-18 serum concentrations with degree of liver steatosis measured using USG. To support results obtained with USG we applied ^1^HMRS. Obtained results demonstrated that hepatopathic children with liver steatosis measured with ^1^HMRS have significantly higher levels of serum IL-18 as well as steatosis score of 2 or 3 in USG than those without liver steatosis in ^1^HMRS. IL-18 serum level above 326.8 pg/mL was finally demonstrated with ROC analysis as an optimal border for differentiation of NAFLD in children. Interestingly elevated IL-18 was accompanied by higher activities of hepatocyte injury enzymes in more advanced steatosis irrespective of diagnostic method with USG or ^1^HMRS. On the other hand there was no effect of steatosis degree on cholesterol and glucose levels, but possible future metabolic problems in children with more advanced steatosis were indicated by significantly higher triglycerides level and insulin resistance.

The studies on adults population have suggested that iron overload plays a significant role in pathogenesis of NAFLD and progression from simple steatosis to nonalcoholic steatohepatitis (NASH) through increasing oxidative stress and altering insulin signaling and lipid metabolism [13]. Since serum ferritin level is related to liver iron storage in NAFLD [39], we included measurement of ferritin in our study. Kowdley at al. [40] demonstrated recently elevated serum ferritin as an independent predictor of advanced hepatic fibrosis among adult patients with NAFLD. Our data confirmed possibility of similar effect in younger population, because of significantly higher ferritin serum concentration among obese NAFLD children. However, in contrast to IL-18 increase of ferritin serum level was not related to liver steatosis, which is contrary to data from adult population that showed predictive role of high serum ferritin as a risk factor for steatosis [41]. Based on these data we can conclude that serum ferritin is related mostly to inflammatory activity in NAFLD patients [42]. However according to results from our study and some literature data, this component of the disease can also be measured using hsCRP [43]. In contrast, elevated IL-18 can reflect degree of steatosis in addition to inflammation and therefore can be recognized as more representative for complexity of NAFLD pathogenesis.

This is a first study in obese children population investigating serum IL-18 concentration. Studies carried out in children may be more reliable compared to those in adults, because of infrequent confounding factors and less advanced disease. Large sample size of obese children is a main strength of our study. Additional value of our study was inclusion of new sensitive imaging technique to assess liver steatosis, such as proton magnetic resonance spectroscopy (*¹*HMRS), providing an opportunity of precise data interpretation [44]. However there are some potential limitations of the study. First of all, we did not perform histological examination to confirm diagnosis in the studied group. According to the ESPGHAN Hepatology Committee guidelines liver biopsy is preferred but imperfect gold standard, because as an invasive procedure it has important limitations in children, including risk of complications, cost, and possible sampling error [45]. Therefore, according to accepted recommendations liver biopsy should be performed only in children in very specific cases of NAFLD suspicion, such as suspected advanced disease, to exclude coexisting diseases, in patients below 10 years of age with elevated ALT activity and before therapeutic intervention [46]. Children included in our study did not meet these criteria; therefore we did not perform a liver biopsy. We still need to validate reliable, noninvasive tests that could be useful to evaluate children with suspected NAFLD and determine disease progression. Another limitation of our study was selection bias, because examined patients were recruited from a tertiary center, which is focused on pediatric hepatology. Therefore children with suspected liver disease were initially referred to the center and then included in the study group.

In conclusion, the study demonstrated association between NAFLD and IL-18 serum concentration in children. Based on this finding we can consider IL-18 as a useful novel noninvasive biomarker for differential diagnosis between obese children with and without NAFLD and prediction of possible metabolic disorders development.

## Figures and Tables

**Table 1:** Analyzed parameters in all obese patients included in the study.

Parameters	NAFLD median; Q1–Q3 (*n* = 39)	Non-NAFLD median; Q1–Q3 (*n* = 69)	*p*
Gender, M/F	33/6	51/18	NS
IL-18 (pg/ml)	335; 257–493	299; 219–383	0.034
BMI (kg/m^2^)	29; 27–34	27; 25–31	0.007
Waist (cm)	100; 94–108	93; 86–103	0.008
ALT (IU/l)	64; 50–101	26; 19–33	0.000
AST (IU/l)	39; 32–52	25; 21–30	0.000
GGT (IU/l)	30; 23–45	18; 14–23	0.000
Bilirubin (mg/dl)	0.6; 0.5–0.9	0.6; 0.45–0.7	NS
Total cholesterol (mg/dl)	177; 153–216	174; 145–190	NS
HDL-C (mg/dl)	44; 39–51	46; 38–54	NS
LDL-C (mg/dl)	97; 79–143	99; 78–116	NS
TG (mg/dl)	132; 85–173	108; 85–142	NS
Glucose (mg/dl)	93; 85–99	91; 86–96	NS
Insulin (*μ*IU/ml)	18; 14–22	15; 11–18	0.011
HOMA-IR	4.2; 3.2–5.8	3.2; 2.1–4	0.005
Uric acid (mg/dl)	6.5; 5.5–7.4	5.7; 4.8–6.6	0.023
hsCRP (mg/l)	0.9; 0.7–1.8	1.3; 0.6–2	NS
Ferritin (ng/ml)	61; 50–105	52; 34–65	0.005
A1AT (g/l)	1.3; 1.2–1.5	1.4; 1.2–1.5	NS
USG grade of steatosis	2; 1–3	1; 0-1	0.000
^1^HMRS (r.u.)	163; 106–210	80; 40–148	0.000

**Table 2:** Correlation (Spearman) between IL-18 serum concentrations and selected parameters.

	*r*	*p*
ALT	0.20	**0.036**
AST	0.21	**0.032**
GGT	0.23	**0.016**
Bilirubin	−0.13	0.185
Total cholesterol	−0.02	0.811
HDL-C	−0.08	0.399
LDL-C	−0.02	0.841
TG	0.21	**0.027**
Glucose	0.11	0.253
Insulin	0.07	0.462
HOMA - IR	0.08	0.396
Uric acid	−0.04	0.704
hsCRP	0.36	**0.014**
Ferritin	0.05	0.599
A1AT	−0.05	0.588
USG grade of steatosis	0.19	**0.044**
1HMRS	0.12	0.252
BMI	−0.12	0.219
Waist	−0.29	**0.009**

**Table 3:** Comparison of obese children without liver steatosis (*n* = 19) and with advanced liver steatosis (*n* = 42) evaluated in USG according to Saverymuttu et al.

Parameters	No liver steatosis USG grade 0 median; Q1–Q3 (*n* = 19)	Advanced liver steatosis USG grade 2-3 median; Q1–Q3 (*n* = 42)	*p*
Gender, M/F	12/7	36/6	NS
IL-18 (pg/ml)	279; 198–320	335; 249–443	0.027
BMI (kg/m^2^)	27; 25–30	28; 27–33	NS
Waist (cm)	90; 83–98	99; 93–107	0.014
ALT (IU/l)	20; 16–31	48; 36–77	0.000
AST (IU/l)	23; 20–29	32; 26–45	0.000
GGT (IU/l)	18; 13–23	27; 21–35	0.000
Bilirubin (mg/dl)	0.6; 0.5–0.7	0.6; 0.4–0.85	NS
Total cholesterol (mg/dl)	164; 137–182	170; 153–191	NS
HDL-C (mg/dl)	47; 39–56	42; 37–50	NS
LDL-C (mg/dl)	92; 73–111	96; 79–121	NS
TG (mg/dl)	86; 65–116	120; 88–167	0.004
Glucose (mg/dl)	90; 83–96	95; 90–99	NS
Insulin (*μ*IU/ml)	14; 8–17	17; 12–21	NS
HOMA-IR	3.2; 1.9–3.7	3.9; 3–5.4	0.020
Uric acid (mg/dl)	5.9; 4.8–7	6.7; 5.6–7.3	NS
hsCRP (mg/l)	1.1; 0.7–2.2	1.1; 0.5–1.7	NS
Ferritin (ng/ml)	54; 29–66	58; 46–85	NS
A1AT (g/l)	1.4; 1.2–1.6	1.3; 1.2–1.4	NS
^1^HMRS (r.u.)	38; 17–61	172; 123–216	0.000

**Table 4:** Comparison of obese children with (*n* = 72) and without (*n* = 20) liver steatosis diagnosed with measurement of intrahepatic lipid content using ^1^HMRS.

Parameters	No liver steatosis in ^1^HMRS median; Q1–Q3 (*n* = 20)	Liver steatosis in ^1^HMRS median; Q1–Q3 (*n* = 72)	*p*
Gender, M/F	12/8	58/14	NS
IL-18 (pg/ml)	275; 205–340	342; 249–455	0.014
BMI (kg/m^2^)	29; 27–32	28; 26–32	NS
Waist (cm)	96; 88–104	94; 90–105	NS
ALT (IU/l)	25; 16–45	37; 26–63	0.022
AST (IU/l)	24; 21–33	30; 25–39	0.013
GGT (IU/l)	17; 12–23	23; 18–31	0.016
Bilirubin (mg/dl)	0.7; 0.5–1.2	0.6; 0.4–0.8	NS
Total cholesterol (mg/dl)	172; 142–182	176; 150–192	NS
HDL-C (mg/dl)	50; 42–52	46; 39–55	NS
LDL-C (mg/dl)	92; 78–114	97; 78–121	NS
TG (mg/dl)	90; 61–120	115; 88–164	0.008
Glucose (mg/dl)	90; 85–95	92; 85–96	NS
Insulin (*μ*IU/ml)	14; 8–18	15; 12–20	NS
HOMA-IR	3; 1.9–4	3.6; 2.8–4.6	NS
Uric acid (mg/dl)	5.7; 4.8–7	6; 5–6.8	NS
hsCRP (mg/l)	1; 0.7–2	1.3; 0.6–1.9	NS
Ferritin (ng/ml)	55; 29–73	57; 45–86	NS
A1AT (g/l)	1.4; 1.2–1.6	1.3; 1.2–1.5	NS
USG grade of steatosis	0.5; 0-1	1.5; 1-2	0.000

**Table 5:** Analysis of AUC for IL-18, ALT, AST, GGT, and TG and their combination to detect children with liver steatosis in ^1^HMRS.

Parameters	AUC	SE	95% CI (AUC)	*p* (AUC = 0,5)	*p* (AUC individual versus combined)
IL-18	0,6802	0,0653	(0,552–0,808)	0,0058	0,263
ALT	0,6681	0,0787	(0,514–0,822)	0,0327	0,253
AST	0,6830	0,0769	(0,532–0,834)	0,0173	0,279
GGT	0,6767	0,0792	(0,521–0,832)	0,0257	0,324
TG	0,6944	0,0614	(0,574–0,815)	0,0015	0,316
Combined	0,7826	0,0532	(0,678–0,887)	<0,0001	-

## Abbreviations

ALT: Alanine aminotransferase
A1AT: Alpha-1-antitrypsin
AUC: Area under curve
AST: Aspartate aminotransferase
BMI: Body mass index
CRP: C-reactive protein
DILI: Drug-induced liver injury
GGT: Gamma glutamyltransferase
HBV: Hepatitis B virus
HCV: Hepatitis C virus
HDL: High-density lipoprotein
hsCRP: High-sensitive C-reactive protein
HOMA-IR: Homeostasis model assessment of insulin resistance
IR: Insulin resistance
IFN-gamma: Interferon-*γ*
IL-18: Interleukin-18
LDL: Low-density lipoprotein
MS: Metabolic syndrome
NAFLD: Nonalcoholic fatty liver disease
NASH: Nonalcoholic steatohepatitis
*¹*HMRS: Proton magnetic resonance spectroscopy
ROS: Reactive oxygen species
ROC: Receiver operating characteristics
TG: Triglycerides
USG: Ultrasonography
WBC: White blood cell.
